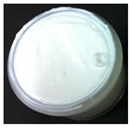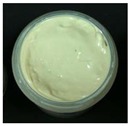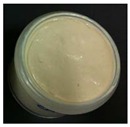# Antimicrobial Silver Chloride Nanoparticles Stabilized with Chitosan Oligomer for the Healing of Burns

**DOI:** 10.3390/ma9040215

**Published:** 2016-03-23

**Authors:** Yun Ok Kang, Ju-Young Jung, Donghwan Cho, Oh Hyeong Kwon, Ja Young Cheon, Won Ho Park

**Affiliations:** 1 Department of Nano Manufacturing Technology, Nano Convergence Mechanical Systems Research Division, Korea Institute of Machinery and Materials, Daejeon 34103, Korea; ok2733@gmail.com; 2 Department of Veterinary Medicine, Chungnam National University, Daejeon 305-764, Korea; jyjung@cnu.ac.kr; 3 Department of Polymer Science and Engineering, Kumoh National Institute of Technology, Gumi 39177, Korea; dcho@kumoh.ac.kr (D.C.); ohkwon@kumoh.ac.kr (O.H.K.); 4 Department of Advanced Organic Materials and Textile System Engineering, Chungnam National University, Daejeon 305-764, Korea; alranim@nate.com

**Keywords:** Silver chloride nanoparticles, Chitosan oligomer, Burn wound healing, Antibacterial activity

## Abstract

Recently, numerous compounds have been studied in order to develop antibacterial agents, which can prevent colonized wounds from infection, and assist the wound healing. For this purpose, novel silver chloride nanoparticles stabilized with chitosan oligomer (CHI-AgCl NPs) were synthesized to investigate the influence of antibacterial chitosan oligomer (CHI) exerted by the silver chloride nanoparticles (AgCl NPs) on burn wound healing in a rat model. The CHI-AgCl NPs had a spherical morphology with a mean diameter of 42 ± 15 nm. The burn wound healing of CHI-AgCl NPs ointment was compared with untreated group, Vaseline ointment, and chitosan ointment group. The burn wound treated with CHI-AgCl NPs ointment was completely healed by 14 treatment days, and was similar to normal skin. Particularly, the regenerated collagen density became the highest in the CHI-AgCl NPs ointment group. The CHI-AgCl NPs ointment is considered a suitable healing agent for burn wounds, due to dual antibacterial activity of the AgCl NPs and CHI.

## 1. Introduction

Antibacterial materials could be used in food packaging or handling, medical tools, hospitals where people are more vulnerable to infections, possible areas like bathrooms where personal hygiene is important, air and water filtration, *etc.* Among various materials known to be effective against a variety of bacteria, silver (Ag) compounds (including elemental silver, silver oxide, silver halide, *etc.*) are unique materials due to their powerful antibacterial activities against nearly 650 bacteria strains. In addition, Ag compounds are well-known and have already been used in many biological and medical fields, such as biosensors, wound healing materials, dental resin composites, and cancer therapeutics [1,2].

Traumatic wounds including burn wound occur frequently in skin loss, and bacterial infections often result from heavy contamination. There are many dressings and creams available to clinicians for use in treating burn wounds. Numerous agents are valued for their antibacterial activity, which can prevent colonized wounds from becoming infected, thereby assisting the wound to heal. Wound infection is one of the main problems and serious consequence in severe burns or extensive skin loss since bacteria produce leukocidin and tissue-destroying enzymes that further impair healing. As a result, wound infections alter and delay normal the wound healing mechanism. One of the approaches for treating a wound infection is the use of wound dressings containing antibacterial materials with a broad-spectrum of activity. Ag compounds have been widely found in wound dressings in various forms, such as elemental Ag (Ag metal, and nanocrystalline Ag), inorganic compounds (silver oxide, silver phosphate, silver chloride, silver sulfate, silver calcium-sodium phosphate, silver zirconium compound, and silver sulfadiazine), and organic complexes (silver zinc allantoinate, silver alginate, and silver carboxymethylcellulose) [3,4,5]. Recent evidence suggests that the antibacterial mechanism is due to the release of Ag^+^ or Ag^0^. In addition, this antibacterial effect accelerates wound healing [6,7]. The slow release of Ag ions is required for a continual bactericidal concentration of Ag ions in the wound. Silver chloride (AgCl) is colorless and its low solubility product ensures a long life and a slow release of silver ions for antibacterial property [8,9]. Indeed, there have been some reports on using AgCl for antibacterial agent in wound dressing [10,11,12].

Chitosan, a β-1,4-linked polysaccharide of glucosamine (2-amino-2-deoxy-β-D-glucose) with lesser amounts of N-acetylglucosamine, is a natural non-toxic biopolymer derived by deacetylation of chitin [13]. Chitosan oligomer (chitooligosaccharide, CHI) is easy to prepare by the acidic or enzymatic partial hydrolysis of chitosan. It has been reported that lower oligomers of chitosan are water-soluble and biologically active, through their solubility and activities are dependent on average degree of polymerization (DP) and the degree of deacetylation (DD) [14,15]. Chitosan and chitosan oligomer have pronounced antibacterial effects because of the presence of amino groups [16,17]. Many studies have also been carried out on the use of chitosan and its derivatives as a wound healing accelerator by enhancing the functions of inflammatory cells, and there is good evidence that chitosan can beneficially influence every separate stage of wound healing [18,19,20,21,22]. Chitosan and chitosan oligomer are currently being explored as novel tools for wound and burn dressings, because of their immunostimulating, hemostatic, antibacterial, nontoxic, biocompatible, and biodegradable properties [23].

In previous study [24], AgCl NPs stabilized with CHI were prepared by green synthesis. The effect of the CHI, which was used as a resource of Cl ions and a stabilizing agent, on the formation reaction of the AgCl NPs was investigated. It was confirmed that the Cl ions remained around ammonium group in CHI molecule since the CHI was made by acidic hydrolysis using hydrochloric acid. Ag ions readily reacted with the Cl ions during the formation of AgCl NPs. The synergistic effect of CHI-AgCl NPs on the antibacterial activity was confirmed to be because it was prepared by the combination of CHI and AgCl NPs, known antibacterial materials. However, further studies on accelerating effect of the *in vivo* wound healing by antibacterial CHI-AgCl NPs have not been studied.

The present study was carried out to give an example of an effective burn wound healing agent based on synergistic effect from using both AgCl and CHI. We synthesized CHI-AgCl NPs using water-soluble chitosan oligomer by an environment-friendly method. Furthermore, the CHI-AgCl NPs ointment was prepared to evaluate the healing effect for burn wound using rat model with the expectation of applications in pharmaceutical and biomedical fields.

## 2. Materials and Methods

### 2.1. Preparation of CHI-AgCl NPs

Oligomeric chitosan (CHI, DD = 87%) was supplied by Hyosung Co. (Korea), and its composition is as follows: dimer 2.31, trimer 12.53, tetramer 15.11, pentamer 13.59, hexamer 8.86, heptamer 6.46, octamer 8.87, and nonamer or higher 32.27 mol %. Cl ions (Cl^-^) remained in CHI molecule because CHI was made by acidic hydrolysis using chitosan in hydrochloric acid. CHI was used as a resource of Cl ions and stabilizing agent for preparing AgCl NPs. Distilled water was used as solvent in all the syntheses to provide benign environmental conditions in this system. In a typical preparation, 0.5 mL of 0.1 M AgNO_3_ solution was added to 60 mL of 5% (w/v) CHI solution. After complete dissolution, the mixture was reacted in a three-necked glass-stopper flask fitted with a double-walled spiral condenser to prevent evaporation and heated to 70 °C for 300 min. All solution components were purged with nitrogen gas to eliminate oxygen. After the formation of CHI-AgCl NPs, the suspension was freeze-dried immediately at –85 °C to produce the powder CHI-AgCl NPs. A series of experiments were performed to obtain transparent CHI-AgCl NPs by varying the order of reactants and the reaction temperature.

### 2.2. Characterization of CHI-AgCl NPs

A UV-Vis spectrophotometer (Shimadzu, UV-2450, Tokyo, Japan) was used to record absorption spectra in the suspension of CHI-AgCl NPs using a cell with path length of 1.0 cm. The morphology of CHI-AgCl NPs was observed using a transmission electron microscope (TEM) (EM 912 OMEGA, ZEISS, Jena, Germany). The samples for TEM observation were prepared by spotting a few microliters of the suspension of synthesized CHI-AgCl NPs onto a holey carbon TEM grid followed by drying before putting them into the TEM sample chamber.

### 2.3. Preparation of Ointments

Three ointments for *in-vivo* burn wound healing were prepared from Vaseline, CHI, and CHI-AgCl NPs powder. The burn wound healing effect of CHI-AgCl NPs against CHI alone was examined, and Vaseline was used as a base component of the ointment. The composition of each ointment for burn wound healing is described in Table 1. The CHI and CHI-AgCl NPs powder were dissolved in distilled water to obtain 10% (w/v) aqueous solutions at room temperature, which were used in the water phase. Twelve grams of Vaseline, 12 g of stearyl alcohol and a 4 g of surfactant (Cremophor RH40, HCO-40, SIGMA, Darmstadt, Germany) were heated at 75 °C and mixed with the water phase solution (40 mL) at 75 °C to obtain an emulsion. Uniform ointments were obtained after slow cooling of the emulsion.

### 2.4. Burn Wound Model

The experiment was approved by the institutional committee for animal care in laboratory research. For all experiments, four-week-old Sprague-Dawley rats were housed and bred at the experimental animal center of Chungnam National University. The animals were provided with a commercial diet and water *ad libitum* under temperature-, humidity-, and lighting-controlled conditions (22 ± 2 °C, 55 ± 5%, and a 12:12-h light–dark cycle, respectively). Procedures involving animals and their care were conducted in accordance with our institutional guidelines, which comply with international laws and policies [25]. To induce burns with skin damage, a slightly modified soldering iron with a flat contact area of 28.3 mm^2^ (AD = 6 mm) was made. Before creation of the burn wound, rats were anaesthetized by Tiletamine plus Zolazepam, (30 mg/kg + 10 mg/kg) according to body weight. Hair on the dorsal side of the rats was removed, and a burn wound was inflicted by placing the circular iron disc (heated to 95 °C) over the dorsal side for 20 s. Second-degree burns without cellular and tissue structure in the dermis were observed by sections stained with hematoxylin and eosin (H&E). Animals were divided after 7 days of acclimation in the cage, and then assigned equally (n = 30) to one of the following groups: Group 1-untreated controls dressed with gauze; Group 2-treated with the Vaseline ointment with gauze; Group 3-treated with CHI ointment with gauze; and group 4-treated with CHI-AgCl NPs ointment with gauze. Small amounts of ointment were applied everyday for 21 days. The sample tissue was enucleated after treatment 1, 3, 7, 14, and 21 days before sacrifice. The skin tissue for histopathological analysis was fixed in 10% buffered formalin, subsequently dehydrated, and embedded in paraffin. The tissue paraffin was cut into 5 µm sections. Fixed sections were then stained with H&E and Masson’s trichrome staining (MT).

### 2.5. Analysis of Blood Counts

The biochemical analysis of the whole blood was performed to confirm the healing process. The blood sample was obtained from the abdominal aorta of animals before sacrifice. After being placed in a serum tube containing the EDTA, and the tube was shaken with an orbital shaker. Complete blood counts were measured using an automatic blood chemical analyzer (MS9-5, CARESIDE CO. LTD, Seongnam, Korea).

## 3. Results and Discussion

### 3.1. Characterization of CHI-AgCl NPs

**Figure 1:**
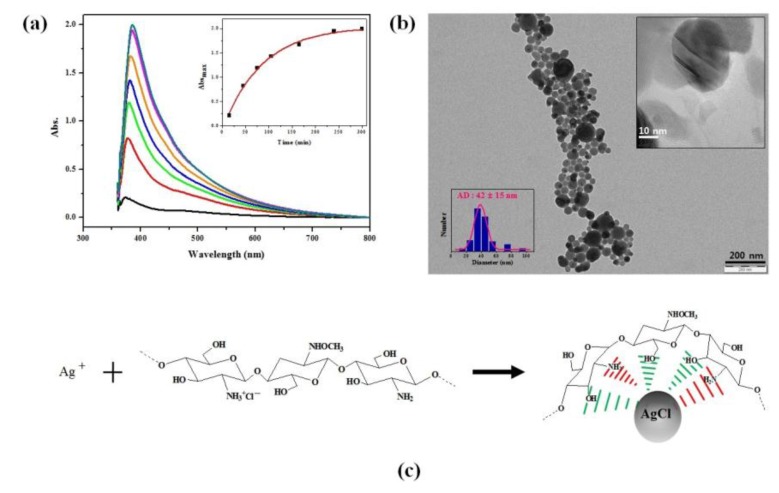
(**a**) UV-Vis spectra of CHI-AgCl NPs dispersion with reaction time (the inset shows the change in absorbance at 400 nm with reaction time); (**b**) TEM image and the corresponding particle size distribution (inset) of the CHI-AgCl NPs; and (**c**) a proposed mechanism for the formation of CHI-AgCl NPs.

The formation of CHI-AgCl NPs was confirmed easily by both the color change from yellow to yellowish brown with increasing reaction time and the strong surface plasmon resonance (SPR) peak around 400 nm due to the formation of spherical AgCl NPs [12]. Figure 1a shows some typical UV-Vis spectra of the suspension with CHI-AgCl NPs for the different reaction times. A strong SPR peak was observed at around 400 nm, which is a characteristic of spherical faceted AgCl NPs. A red shift of the absorption maximum was observed with increasing reaction time. The inset graph in Figure 1a shows the change in absorbance at λ = 400 nm of the CHI-AgCl NPs suspension with the reaction time for 300 min. To further investigate the morphology of CHI-AgCl NPs, the sample was analyzed with a field emission scanning electron microscope. There is a roughly spherical CHI-AgCl NPs, as seen in Figure 1b. The size of the roughly spherical CHI-AgCl NPs ranges between 30 and 50 nm, and their average diameter is 42 ± 15 nm (inset image in Figure 1b). It was assumed that the formation mechanism of AgCl NPs occurred in two steps. The mechanism on the formation of CHI-AgCl NPs is illustrated in Figure 1c. Firstly, the Ag ions readily reacted with Cl ions, which remained around the ammonium group of CHI after the acidic hydrolysis by hydrochloric acid (HCl). Secondly, small AgCl NPs were stabilized with amino and hydroxyl groups in CHI and underwent growth to large particles [26,27,28,29,30].

### 3.2. A Clinical Pathology Study

**Figure 2:**
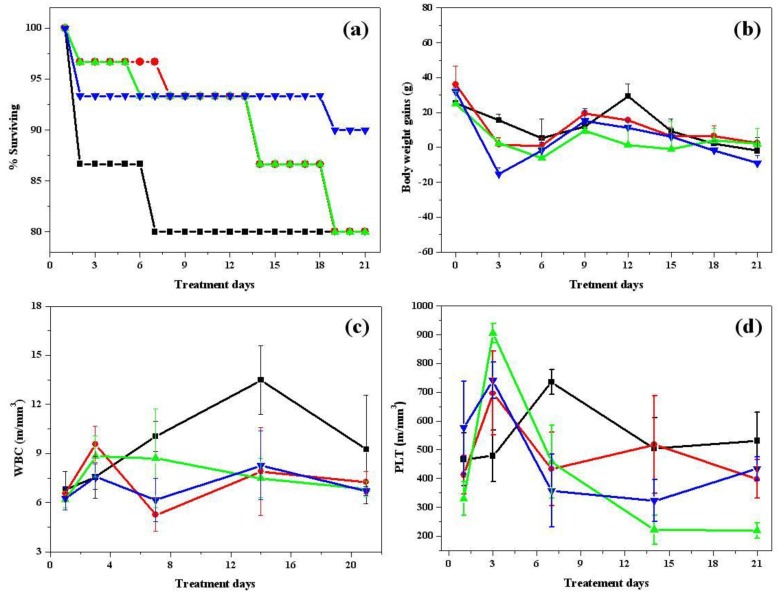
The *in-vivo* clinical pathology study of untreated (■), Vaseline (●), CHI (▲) and CHI-AgCl NPs ointment (▼) on: survival curves (**a**); body weight gains (**b**); concentration of white blood cells (WBC) (**c**); and concentration of platelet (PLT) (**d**).

A clinical pathology study was conducted during *in-vivo* burn wound treatment to determine the effects of burn trauma on skin that was infected and dehydrated after sustaining injury. The survival curves were found to be significantly different between the ointment-treated groups (Groups 2–4) and untreated group (Group 1). The survival rates of the CHI-AgCl NPs ointment group were 90%, but those of the untreated, Vaseline, and CHI groups were 80% at 21 days (n = 30). In all groups of animals, most of the fatalities occurred between Day 1 and 6 because of the post-burning infection (Figure 2a). In particular, the surviving rate was highest in the CHI-AgCl NPs ointment group in comparison with other ointment treated groups (Groups 2 and 3), making it clear that the CHI-AgCl NPs ointment had prevented the infection and improved the burn wound healing. The body weight gain and components of blood were determined for the clinical pathology study during the burn wound healing process. The body weight gain was compared in Figure 2b. On early treatment days until Day 3, the body weight gain was decreased for all groups. There was no significant difference in all groups during treatment days. White blood cells (WBC) are involved in defending the body against both infectious disease and foreign material. Although the normal range of a white blood cells was 6.60 to 12.6 m/mm^3^, the amount of WBC was somewhat increased in all of the untreated and treated groups after sustaining injury [31]. It was still increased in the untreated group with increasing treatment days, but those of the other treated groups were decreased. The variation of WBC in CHI-AgCl NPs treated group (Group 4) was the lowest of all groups, which indicated that the CHI-AgCl NPs ointment reduced the infection, resulting in accelerating the burn wound healing. The amount of platelets was increased in the all groups during early-treatment days because the burn wound was created, and was reduced thereafter with increasing treatment days (Figure 2c,d).

### 3.3. Histological Analysis

**Figure 3:**
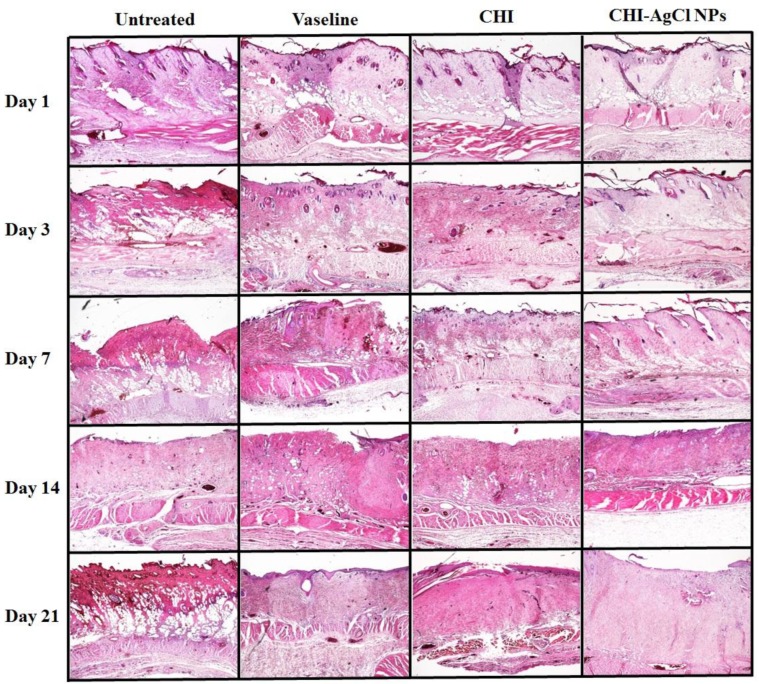
Evaluation of histopathology in the healing effect of ointments on burn induced skin damage using the H&E staining. The photographs were taken at an original magnification of ×40.

The healing pattern of the burn wounds was studied to examine the histology for the untreated, Vaseline, CHI, and CHI-AgCl NPs groups on Days 1, 3, 7, 14, and 21 using H&E staining method (Figure 3). Generally, the injury initiates inflammatory phases for healing [32]. Therefore, it is difficult to assess whether the inflammatory response is part of the normal healing process or an effect of the material during the early stages of wound healing. For the untreated group, the epidermis is completely destroyed and interrupted, and the tissue of the wound is filled by necrotic material, bulla, and infiltrated inflammation until Day 21. Hyperkeratosis was also observed on Days 7 and 14. The Vaseline-treated group was shown to be similar to the untreated group. At 21 days, however, the Vaseline-treated group exhibited less infiltrated inflammation than untreated group, but not bulla. In the CHI ointment-treated group, the number of bulla was gradually decreased, and infiltrated inflammation was not observed in comparison to the Vaseline-treated group. In contrast, the CHI-AgCl NPs ointment-treated group was shown to have little infiltrated inflammation, and the underlying area showed fibrosis with proliferation of fibroblasts that were placed in the granulation tissue, and started to fill up the regenerated tissue from Day 14. After 21 days, the epidermis was filled with mature granulation tissue like normal tissue, confirming the accelerated burn wound healing by the improved antibacterial activity of the AgCl NPs stabilized with CHI.

### 3.4. Evaluation of Collagen Percentage

**Figure 4:**
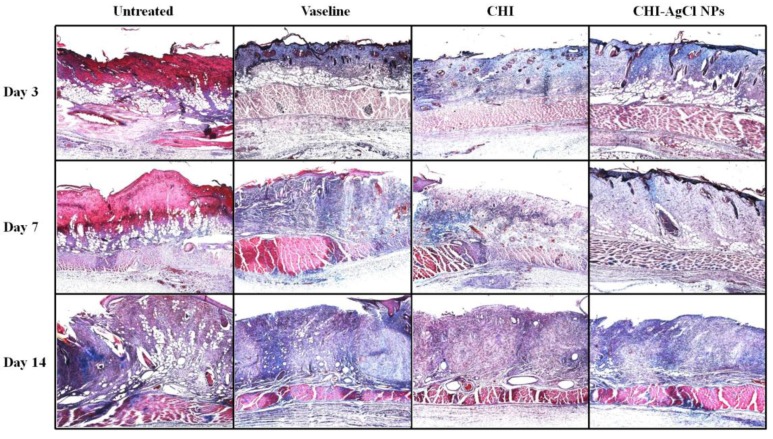
Evaluation of histopathology in the healing effect of ointment on burn induced skin damage using the Masson’s trichrome staining. The photographs were taken at an original magnification of ×40.

**Figure 5:**
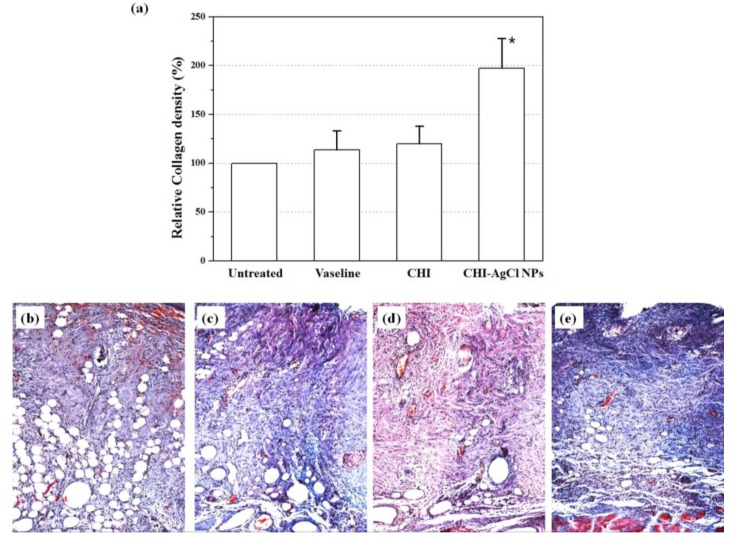
(**a**) The relative collagen density in burn induced skin damage at treatment Day 14. Evaluation of histopathology in the healing effect of untreated (**b**); Vaseline (**c**); CHI (**d**); and CHI-AgCl NPs (**e**) ointment using MT staining. The photographs were taken at an original magnification of ×100. The data are mean ± SD of 10 independent experiments. * indicates data with a statistical significance (*p* < 0.05) compared with the untreated and CHI-AgCl NPs ointment-treated groups.

Collagen is the main structural protein component of connective tissue, and is mainly found in skin. Therefore, it is desirable to observe the regenerated collagen in damaged skin during the healing process. To observe the collagenous components, the wounds with or without treated ointment were stained using a Masson’s trichrome staining method. Figure 4 shows representative photomicrographs of wound healing on Days 3, 7 and 14. The collagenous components were stained in blue, and the cytoplasm appeared in varying shades of red. It was precisely observed that the necrosis was filled in the regenerated tissue in untreated group (Group 1), whereas the ointment-treatment (Groups 2–4) influenced the regeneration of collagen for burn wound healing. For an accurate description, the density of collagen at 14 days was determined using an image analyzer (Nikon, Japan), as shown in Figure 5. The relative collagen density was increased significantly in the ointment-treated groups, compared to that of the untreated group. Among them, the CHI-AgCl NPs-treated group (Group 4) showed the highest collagen density, suggesting increased collagenase activity by the dual antibacterial activity of the AgCl NPs stabilized with CHI. The data were represented as the mean ± standard deviation (SD) of 10 independent experiments, and difference was significant at *p* < 0.05 compared with the untreated, Vaseline, and CHI groups.

## 4. Conclusions

In view of the rapid progress of application of nanomaterials in bioengineering fields, environmentally friendly methods should be required since the common methods generate toxic and biological hazards. The CHI-AgCl NPs was successfully obtained by a simple and environmentally benign method that used water and CHI as a biomaterial. The CHI is fundamental in the formation and stabilization of well-dispersed AgCl NPs with a mean diameter of 42 ± 15 nm. The burn wound treated with CHI-AgCl NPs ointment showed the highest percent of survival, and a reasonable number of white blood cells because of the prevention of infection in wound healing. The collagenous components were more regenerated in the CHI-AgCl NPs ointment group than the other treated groups. It was demonstrated that the CHI-AgCl NPs were more effective as a wound-healing accelerator because of improved antibacterial activity of CHI exerted by the AgCl NPs. Consequently, CHI-AgCl NPs ointment is considered a promising material for burn wound healing.

## Figures and Tables

**Table 1:** The composition of each ointment for *in-vivo* burn wound healing in the rats.

Ointment Composition	Vaseline	CHI	CHI-AgCl NPs
Oil phase	12 g Vaseline, 12 g Stearyl alcohol, 4 g Cremophor RH40		
Water phase (40 mL)	–	4 g CHI	4 g CHI-AgCl NPs
Optical images